# Towards socially robust policy modelling: scoping review of public involvement in computational policy modelling

**DOI:** 10.1186/s12961-026-01473-6

**Published:** 2026-03-11

**Authors:** Ellen Stewart, Natalie Dewison, Edit Gedeon, Clementine Hill-O’Connor

**Affiliations:** University of Glasgow, Glasgow, UK

**Keywords:** Computational modelling, Public involvement, Socially robust research

## Abstract

Computational policy modelling appeals to policymakers seeking to understand potential outcomes of policy decisions, yet there are long-standing concerns about the “social robustness” of the knowledge it generates. A key route to socially robust policy modelling is the active involvement of publics as partners, and not only subjects, of modelling; yet the technical and abstracted nature of the modelling process poses particular challenges for conventional involvement practice. This scoping review of published computational modelling papers which report public involvement explores both practical elements of involvement in modelling and the tacit or explicit justifications authors offer for involving publics. We found a preponderance of professional stakeholders over “lay” publics and a bifurcation between informal feedback and highly structured input. We conclude that approaches to public involvement in computational policy modelling should seek ongoing dialogic involvement across the stages of the modelling process, be more attentive to power dynamics in the involvement process, and consider how involvement can be inclusive of diverse publics.

**Supplementary Information:**

The online version contains supplementary material available at 10.1186/s12961-026-01473-6.

## Introduction

Computational policy modelling – “the computer implementation of algorithms that policymakers can interact with to inform their decision-making” [1] – is a key tool in the contemporary relationship between evidence and policy. Modellers can offer decision-makers “advanced tools [that] attempt to capture the more dynamic and complex aspects of societal or economic development by performing computer-based modelling simulation” [2]. In the United Kingdom, influential examples include a group of microsimulation models used by government departments, research institutes and think tanks to simulate outcomes of different potential policy decisions [3, 4]. Policy modelling can show how different policy interventions are likely to change the distribution of different outcomes, and may also be a tool for broader public engagement with policy [5]. Scholarly literature demonstrates first that algorithmic models are appealing to policymakers [1, 2, 6, 7], and second, that they can lack transparency and comprehensibility to non-experts [8, 9]. These two sets of debates are interlinked: concerns about transparency are heightened by evidence that models are influential. Long-standing concerns about the transparency and accountability of modelling (see, for example, [10]) are only amplified in the era of big data and computational analysis: modellers can draw on vast amounts of data, without interaction with the people it represents, and the people represented having little or no knowledge about the way their data are being used [11]. While there are examples of participatory approaches within broader systems science (see, for example, [12–14]), Staniszewska et al. argue that *computational* modelling projects rarely involve publics in their research, and that modellers have “not embraced the potential” of these approaches [9].

This scoping review explores current reported practice for involving publics in the computational modelling research process, on the basis that such involvement is one route to building the transparency and accountability of modelling. Nowotny [15] coined the term “socially robust science” to argue for an approach where publics are active partners in science, and not only research participants. Active partnership is concerned with publics having informed and agentic roles in choices being made by the research team, rather than more passively providing data (for example, by completing a survey), which the research team decide how to interpret, analyse and model [16]. In their framework for involving publics in mathematical and economic modelling, Staniszewska et al. specify the goal of creating what they call “a deliberative knowledge space” within public involvement meetings; referring to a relaxed and respectful space for mutual learning and dialogue between publics and researchers, rather than to more formal structures of mini-publics and deliberative democracy [17]. This assertion of the value of publics as authentic agents in the research process, not as objectified repositories of knowledge to be mined, is ever more critical in a moment where researchers are advocating for human survey respondents to be replaced by generative artificial intelligence [18, 19]. However, active/passive roles are the outcome of a set of relational practices and research choices. There remain significant variations of practice within these broadbrush and essentially subjective distinctions, which might show up differently for different fields and projects [20].

To understand how modellers are currently reporting their efforts towards public involvement, we conducted a scoping review of current practice in public involvement within policy-relevant computational modelling research. We are a team of health researchers, but on the basis of our expectation that literature would be relatively small, we searched for and included examples of public involvement in computational modelling across all policy-relevant topics. Different fields of research have different terminology and expectations around public roles in research, but a range of research funders now encourage or mandate researchers to plan for these roles in their research process [21–25]. While our scoping review goes beyond health research, notably being dominated by environmental studies, in this paper we use the language of public involvement as an overarching category, given our own expertise and the journal’s audience. However, in order to understand currently reported practice in modelling research, our scoping review takes in a more expansive set of approaches, which may not meet the definition of “active partnership”. This acknowledges that public involvement in modelling is not well-established [9]. Operating with a more expansive definition, we compare a diversity of approaches to involving publics (broadly defined) along four axes: rationale, who is involved, stage of modelling process and methods of input. On the basis of this corpus, we consider how well current approaches serve the goal of more active roles for publics in computational modelling research, as a route to social robustness.

## Methods

We conducted a scoping review guided by the methodological framework of the Joanna Briggs Institute. Following this framework, a Population, Concept, Context (PCC) framework was used to define the review question, search strategy and the inclusion and exclusion criteria. In practice, and in response to difficulties in identifying relevant literature in this dynamic and emergent area of practice, we also supplemented this approach in ways we describe below.

### Formal search strategy

The search strategy was developed in consultation with an information scientist at the University of Glasgow and the broader research team. Keywords relating to the PCC framework were used to determine the search terms, which were subsequently applied to two databases: Scopus and Web of Science. Both search strings used can be found as part of the Supplementary Materials.

In the process of testing and amending search strings, it became clear that the topic required more iterative approaches. In addition to the database searches, we hand-searched the reference lists of included papers for relevant articles. We circulated a request to the members and the Advisory Boards of two policy modelling consortia (SIPHER, and Policy Modelling for Health) [removed for peer review] to ask for suggestions of authors or papers to pursue. All of these additional suggestions, from reference searching, our networks, and from process tracing, were screened by the same process as for the list generated through database searching.

### Inclusion and exclusion criteria

To be included in the review, studies had to:

1. Describe a specific project or study where policy modelling has been used
2. Describe the specific methods or approaches used to involve publics in the research process (e.g. data generation, data preparation and analysis). Studies that reported public engagement with policy models through public-facing dissemination and communication were also considered, even if these are not active patient and public involvement (PPI) partnerships
3. Be written in English
4. Be published between 2000 and 2024, given the increased awareness of the importance of PPI in research during this period

### Screening and data extraction

**Fig. 1:**
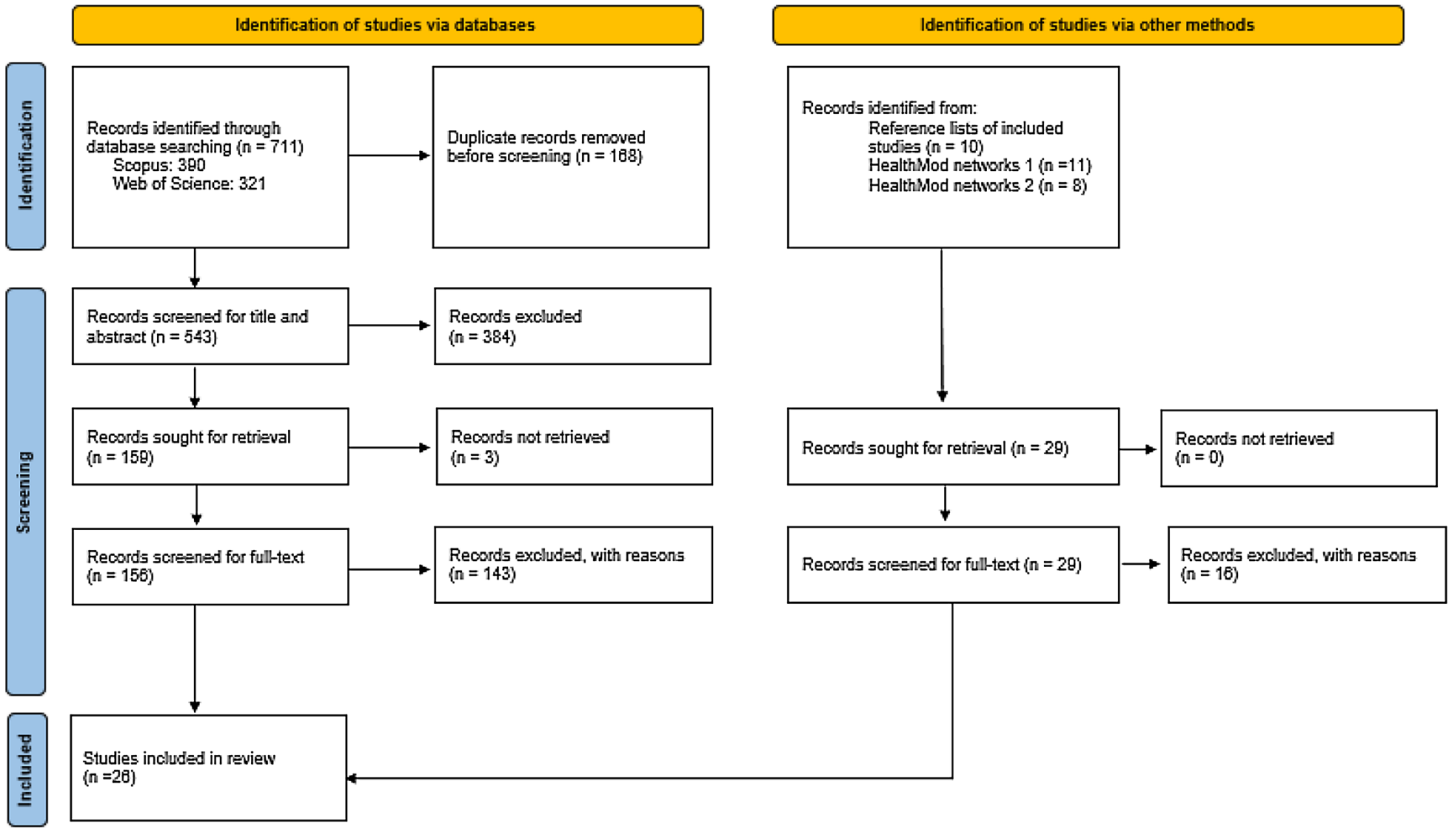
PRISMA flow diagram outlining the article selection process

The article selection process is outlined in the Preferred Reporting Items for Systematic reviews and Meta-Analyses (PRISMA) flow diagram (Fig. 1). All items retrieved through the search process were imported to Rayyan for title and abstract screening. Duplicate search results were removed using Rayyan’s built-in duplicate detection tool. Prior to commencing study selection, EG and ND independently screened the titles and abstracts of a 20% random sample and compared the application of the eligibility criteria to ensure consistency. Disagreements were discussed and resolved. Ambiguous items were passed forward for full-text screening. For full-text screening, ND again independently screened a random sample of 10%. EG screened the remaining available full-texts; the wider team provided a second opinion where ambiguities were identified.

Data extraction was conducted by all four authors using a template developed in Excel. The template was piloted by all four authors. Column headings can be viewed in Supplementary Information. ES and ND conducted a narrative synthesis of the collated results, and we continued to meet and discuss the review as an author team.

Operationalizing criteria 1 entailed excluding some papers closely related to the concerns of this paper, which discussed or described ideas around involvement without describing the actual practice of involving publics in a modelling project [9, 26]. In applying criteria 2, it became clear that there is a significant body of participatory modelling practice which was not captured in this review [5, 13, 27]. What distinguishes this scholarship from the papers captured in this review, is that our search was specifically for papers in which involvement was reported within a *computational* modelling project. Operationalizing criteria 2 was also complicated by the diversity of “publics” engaged, and a lack of reporting of these details. We took an expansive definition of publics as “situational entities” which constitute around a problem of common interest and organize themselves to address it [28]. This included professional stakeholders in some cases, and we reflect below on what this means for concerns about “lay” public involvement.

## Findings

Applying our inclusion criteria resulted in a corpus of 26 papers. While there is a broader hinterland of modelling approaches which can be considered qualitative, in our sample, even papers where the only data inputs were qualitative, were subsequently transposed into numerical form to be modelled (for example, [29]). In terms of topics, of the 26 papers included, the majority were focused on environmental policy (*n* = 17), followed by a cluster of health papers (*n* = 5). The remaining papers concerned urban planning (*n* = 3) and science policy (*n* = 1). Even where papers did meet our inclusion criteria, references to involvement were often brief and lacking key details, reflecting long-standing calls to improve the reporting of public involvement in research [30]. We summarize the content extracted from papers under the following sub-headings: the implied or described rationales for involving publics in modelling research, who is involved, the stage of modelling at which involvement takes place and how involvement takes place (nature of activities and format of input).

### Goals of involvement

We were keen to understand *how* authors described their reasons for having undertaken engagement beyond the academic team, and what *benefits* authors described this yielding. This approach was informed by commonly expressed concerns that within the public involvement literature there has been a focus on practical advice to the detriment of normative debates about rationales [21]. To draw comparisons across papers we used a five-way conceptual distinction common in literature on participatory processes [31]: democratic, consumerist, transformative/emancipatory, substantive and instrumental. While none of the papers in the review explicitly used this language, we have interpreted rationales from descriptions of the stated goals or benefits of activity (Table 1).

**Table 1:** Rationales for involving publics as taken from included papers

Rationale for involvement	Definitions (taken from [31])	Example from reviewed papers
Democratic	“Refers to the right of citizens to have a voice in research that may affect them… implies that PPI in research is the right thing to do and is of intrinsic value”	“Principles of open government can be supported such as participation, transparency and collaboration” [32]
Consumerist	“Relate to neoliberal and economic rights, such as the right to individual choices in the marketplace… includes value-for-money justifications”	“To use collaborative modelling successfully in our context we need to get stakeholders involved with the policy process to, among others, create more understanding of the challenge and support for a policy choice” [33]
Transformative or emancipatory	“Core to this rationale is the amelioration of social inequities… PPI conforming to the transformative rationale has the potential to strengthen disadvantaged and disempowered groups by giving them a chance to speak out on research”	“Involvement of stakeholders helps reconcile their different conflicting interests in wetlands, hence creating a common understanding about the problem under study” [34]
Substantive	“Substantive involvement seeks the inclusion of more diverse, extensive and context-specific bodies of knowledge, in order to increase the quality of the information underlying decision-making”	“No expertise can claim a monopoly of wisdom and competence. Indeed, relevant wisdom is not limited to scientific specialists and public officials. Stakeholders can contribute to knowledge in a number of ways” [35]
Instrumental	“Offers a pragmatic justification for PPI, with the involvement serving as a means to an end… increases the usefulness of the knowledge created… increases the acceptability, legitimacy and ownership of the research process, its outcomes and the solutions of problems studied”	“The Centers for Disease Control and Prevention (CDC) required grantees of the ‘Enhanced Comprehensive HIV Prevention Planning’ (ECHPP) initiative to collaborate with key community and public health stakeholders” [36]

One included paper [37] described nothing that we considered a rationale for processes of involvement, implying that it was simply understood as part of standard practice. The most frequent rationale that we coded was substantive; this related to engaging with broader audiences with the epistemic goal of improving the accuracy and fidelity of the model. For example, in their rationale for involving local residents, Lane et al. [38] referred broadly to the limitations of scientific knowledge (rather than simply existing models or data) pointing to the existence of a knowledge hierarchy in flood risk management which failed to recognize the value of lived experience. The second most common was an instrumental rationale, and papers coded in this category were divided between those where the goal was explicitly satisfying funding or, more often, improving the buy-in of powerholders with a view to enhancing the impact of the model. Only three papers described a more democratic rationale, oriented to the normative right of wider publics to have a say: these included Islam and Kitazawa [34] and Persada et al. [39], both concerned with modelling use of public places. Islam and Kitazawa [34] was also the only paper in which we discerned an emancipatory rationale for involvement, with involvement in the modelling process described as leading to more public action on conservation of wetlands.

### Who is involved

We analysed who was engaged in processes of involvement and (where this information was available) how they were recruited. We coded this information in the following categories: professional stakeholders (i.e. people involved on the basis of their paid work role: 17 papers), community stakeholders (i.e. people in some unpaid representative role in their community: 7 papers), academics (5 papers), model users (generally policymakers: 3 papers), members of the public with specific experiences (3 papers) and the general public (1 paper). Two papers gave no detail beyond referring to the involvement of “stakeholders” [32, 40]. Professional stakeholders were involved owing to their roles within a relevant sector (i.e. public health practitioners in Freebairn et al.’s paper [12]) or within organizations representing those affected by the policy issue being modelled (e.g. farmers as in van Vlient et al. [41]). Policymakers were also included within the category of “professional expertise”, except in four papers where policymakers were explicitly involved as “model users” in relation to the ways that they would use modelling outputs. Overall, in the majority of papers our search for public involvement or engagement yielded papers where the publics in question are *only* professional staff of stakeholder organizations, rather than people in a “lay” capacity.

In most included papers, little or nothing was written about the process of recruitment and selection for the publics involved. One paper referred to using purposive sampling techniques to achieve diversity in perspectives and a range of expertise on the subject area [39]. The perceived (in)capability of the general public and local community members to engage with the modelling process was raised as an issue in two papers, justifying decisions about who was involved. In a European study using “fuzzy cognitive maps” to develop strategic plans for freshwater resource development, the authors opted to involve people who are described as “a level just above… very local stakeholders” (e.g. representatives of farmer organizations rather than individual farmers) as it was argued they would be more familiar with the types of exercises used in workshops [41]. Authors of another European study, developing a decision-making tool for environmental policies, provided a similar justification for stakeholder selection. The authors wanted participants who could “deliver ideas and policy options from their field of expertise [and] be able to think on a higher level of abstraction to deliver context independent knowledge” [35]. The authors did acknowledge that this choice had limitations and that a broader range of perspectives “could have strengthened the knowledge base and the quality of the dialogue in terms of social robustness” (ibid). This perceived trade-off is one we return to in the discussion.

Three papers described participants with specific reference to their experiential knowledge of the policies or issues being modelled. Holtgrave et al. [36] note that the groups involved included people living with HIV – the core focus of their model. Seven papers talked in broad terms about community stakeholders, often based on their geographical proximity to a specific environmental issue. However, in many cases, authors simply referred to engaging “communities”, making it difficult to ascertain exactly who they were, why they were involved and the role they played in the research process.

There were four studies, all focused on environmental issues, within which local people from the affected areas were involved in the modelling process. This included people living in areas affected by frequent flooding [38, 42], groups of individuals living and working in wetlands threatened by degradation, such as farmers, anglers and activists [34], and people displaced by the construction of a hydro-electric dam [29]. One paper [38] stood out for its unusual level of detail in relation to the selection and recruitment of eight participants from the town (a purposefully broad approach to avoid preconceptions of “representativeness”).

### Stage of modelling

We coded included papers for the modelling stage in which they involved publics, broadly following Millington et al. [43] in using sequential categories of conceptualization, formalization, testing and use. Conceptualization was the most common phase in which involvement took place, with typical tasks including identifying the structure of the models and relationships between factors. The advantage of engaging publics in conceptualization processes is that it can be accomplished without requiring mathematical confidence of model formalization: “storylines remain close to the everyday language of stakeholders… quantitative knowledge is not needed” [41]. Indeed, this stage of modelling is one where there is a significant body of experience in participatory approaches (notably, participatory systems mapping), much of which was excluded from this review because it does not go on to report direct contributions to a computational model.

Within reviewed papers, non-academics were far less commonly involved in model formalization, for which tasks included deciding on data sources, deciding on the strength of relationships between factors, and quantifying them. Bluntly, one paper states “stakeholder involvement in the modelling part is often regarded as overly complicated, and involving of lay persons as impossible” [41]. Mathematical aspects of research have been identified by a number of authors as one of the most challenging types of work in which to achieve meaningful public involvement [9, 44]. Nonetheless it is striking that only one of the papers in our review described actively involving members of the public in model formalization [45].

Involvement in testing was more common, albeit more consultative, with researchers demonstrating model runs and asking for feedback. Arguably, involving publics in conceptualization and then testing can constitute involvement in formalization, if the feedback from testing is used to adjust and amend the formalized model. However, this level of detail (examples of feedback being given and acted on) was not reported in any paper included in our review.

Papers coded as “involvement in model use” reported only one-way communication or promotion of the model from researchers or model users to publics. Even where this sought to inform broader publics about important issues, it is not active involvement of publics in the modelling process. For example (in a paper which describes some routes not taken for learning from public perspectives): “In order to enable public participation, the municipality plans to deploy ICT enabled tools including a 3D virtual reality (VR) application available on the Internet. This application will allow urban planners to present their plans to a wide audience and hence raise the awareness among citizens about future developments” [46]. Among papers which reported efforts to build modelling literacy among the general public, Freebairn et al.’s [12] plain language fact sheet and podcast stood out as particularly innovative and considered.

### Methods of input

Broader participatory modelling literature identifies a wide range of methods for gaining input into modelling projects: Voinov et al. [13] list over 20 potential approaches. Although in modelling research these are all described as *participatory* approaches, many of them are standard qualitative research methods rather than approaches to engagement. Our scoping review identified a smaller range of approaches. Interviews and focus groups were two of the more common conventional qualitative methods used [29, 42, 47]. Often these were combined, although in one case, the authors explained that they preferred interviews to avoid power dynamics within a focus group influencing the responses they receive [42].

A majority of papers included methods that we have coded as “workshops”. These vary significantly, and in many cases almost no detail is included about the length, format or structure of the events. Some are described in ways which sound like a conventional academic meeting, with an opportunity for attendees to offer feedback to academics and no sense of how this feedback will be acted on. Others, especially from projects within the participatory tradition, are coded as participatory workshops, which we use to denote a tailored, structured event in which interactive activities are planned to elicit feedback. This sort of workshop tends to take place outside of a conventional academic space, such as in Islam and Kitazawa [34], where seeds were used to undertake scoring of different options with community members. Whilst the line between workshops and focus groups is somewhat blurred, for the purposes of our coding, a key difference was the approach to data generation. Workshops are not generally audio-recorded and transcribed [48]. Data may either be captured via informal note-taking by researchers, or, in the case of participatory workshops, through planned interactive activities, the outcomes of which are recorded in writing. By contrast, in focus groups (as with interviews), the proceedings are audio-recorded, transcribed and analysed by researchers.

Across all the methods, both those reporting what we would consider to be conventional qualitative research and those offering opportunities for involvement to participants, researchers faced the challenge of how to translate narrative textual information into quantified numbers for computational modelling. While we can read this “between the lines” of papers, the type of data which involvement generates is often not extensively reported: papers often referred simply to “feedback” [36, 37]. Alternatively, other papers describe a highly structured approach through which input is generated and used. Several papers outline a method for those involved to generate scenarios for modelling [32, 41]: one offers detailed methods for generating scenarios as “narrative texts formulated in the language of participating stakeholders” [32]. Some papers explicitly report a method to enable the shift from narrative data to scored and quantified perspectives ready for incorporation into modelling. One begins with qualitative interviews and then uses a Delphi study to formalize input [47], while another begins with narrative discussions and then moves to an informal scoring system using seeds and coins [34].

Dilemmas of how to integrate public perspectives alongside other input were evident in a number of papers. This integration requires particular sensitivity to the power dynamics at play where members of the public contribute alongside experts in a conventional meeting scenario. In a paper reflecting on the development of a system dynamics model to explore the implementation and sustainability of a National Health Service (NHS) health programme, the process was overseen by four lay advisors with experience of engaging with programme services [49]. The authors included an evaluation of their experiences, and whilst the lay health advisers felt that they had improved the usefulness and user-friendliness of the tool, they reported that the content was not always clear and described themselves as “translators of information” (to the general public) [49].

## Discussion

The definitional challenges we experienced conducting this review reflect computational modelling and public involvement in research not just as two bodies of literature, but as two distinct epistemic communities of researchers, each with their own beliefs, notions of validity and goals [50]. While there are long-established critiques of reporting standards and terminology in public involvement practice [30, 51], this challenge is particularly heightened in this review. The decision not to report engagement that has been undertaken, or to make brief reference, and not report the ways in which it changed decisions taken by the researchers, reflects a deeper underlying orientation which, while far from unique in academic research, seems pronounced in the field of computational modelling. There are particular challenges in meaningfully engaging non-expert audiences with the exceptionally dense and mathematical terminology and equations that constitute modelling [9, 11] and multiple papers in the review note that, while qualitative systems analysis can be easily communicated to non-modellers, mathematical and algorithmic stages of modelling are more difficult prospects [45].

Given this trickiness, a key finding of our review is that a majority of included papers engaged professionals – policymakers, representatives of stakeholder civil society organizations and even other academics – rather than “lay” publics. Many papers which were initially included because of language such as “coproduction” and “stakeholders”, on closer examination were engaging only professional participants. This reflects an extensive literature within modelling focused on the importance of engaging professional stakeholders and especially potential model users [52]. However, engaging professionals is in many ways an *easier* task than involving lay publics. Professional stakeholders can be recruited through the organizations they work for, are more likely than members of the general public to feel comfortable engaging in abstract technical conversations about policy and research and may have cultivated a professional detachment to issues which can facilitate easy conversations. Nevertheless, involving “lay” publics offers distinct benefits which are unlikely to be replicated working only with professional stakeholders [9, 53]. This is likely to require distinctive methods and approaches to generate an inclusive, respectful and mutually beneficial “deliberative knowledge space” [9] that acknowledges the significant power differential between members of the public and professional researchers. In our own experience of involving publics in modelling projects, enabling them to articulate their personal experiential knowledge *in relation* to modelling conceptualization and formalization, is a daunting task which may, if not very carefully navigated, generate feelings of inadequacy and exacerbate marginalized positions [removed for peer review]. This, as Shaikh et al. [54] argue, can contribute to the “epistemic violence” of narrow modelling approaches to already marginalized participants.

A second key insight of the review process, and a function of our expansive inclusion criterion, was a bifurcation of the included papers between the use of traditional research techniques (interviews, focus groups, surveys) and more participatory approaches (including both participatory research activities and “public involvement” processes within project planning and administration). The key distinction here is between studying publics as research subjects or finding ways to make them active partners in a research project. Our observation is that highly structured approaches to extracting public perspectives often seemed to allow greater translation into (and influence of) computational models. By contrast, papers in which researchers expressed a more normative commitment to public and/or stakeholder involvement, such as Scherer and Wimmer [32] offer minimal reporting of the specific knowledge generated and how it influenced the eventual modelling. Lay publics involved in these more participatory efforts seem to contribute mostly to communicating modelling research [49].

The challenge is to develop approaches which allow lay publics to play active roles in modelling projects, while *also* demonstrably impacting the knowledge generated by models. Nowotny’s (2003) articulation of socially robust research requires an active partnership, and not the mere extraction of public perspectives: she explicitly described the risks of science objectifying societal perspectives. Some of the solutions proposed in included papers – for example, researchers analysing previous consultations on the topic [55] – fall well short of the goals of public involvement advocates. They may be a pragmatic solution to bring additional perspectives into the modelling process, but does not create the dialogic and ongoing relationships with publics required for social robustness. More considered approaches are present in literature: in Lane et al. [38] the authors reflect critically on the experiential knowledge brought to the project by participants including their experience of the locale, their occupational backgrounds and time visiting other places. The eight residents joined a group of academics as one research team, wherein the policy model was not given authority but instead acted as a “critical object” through which knowledge could be negotiated and co-produced [38]. While resource intensive, this exemplifies many of the commitments of social robust modelling: ongoing engagement across the modelling process, careful attention to power dynamics and an approach tailored to the needs of the individuals involved.

## Conclusions

The use of computational modelling to identify policy problems and adjudicate between potential policy solutions is a critical element of shifts towards algorithmic governance [56]. While holding significant promise, this field is also fraught with anxieties about computational models as unaccountable “black boxes” [57]. Involving publics in computational modelling research is one potential route to mitigate these risks. This scoping review of public involvement practice in computational modelling research has identified 26 papers which refer to the involvement or engagement of publics. Our most striking conclusion is that only a far smaller selection of these are, in fact, concerned specifically with lay public (rather than professional) involvement, and the reporting of who has been involved, how they were recruited, and why, is often minimal. Seeking examples of more “active” roles for lay publics, where participants have impactful roles in decision-making and not only in providing data for researchers to process, yielded only one reported study to date [38]. We were additionally struck by the dominance of environmental research within our corpus, suggesting that environmental computational modellers have been quicker to engage publics in the research process than their counterparts in health research.

Computational modelling is an influential method with remarkable potential to “generate proactive pathways for change, or alternatively risk perpetuating the status quo” [5]. As policy modelling techniques become more computationally sophisticated, and big datasets more expansive [11], it is imperative that researchers committed to the tenets of public involvement stay engaged with the field. A more “socially robust science” [15] relies upon ongoing, relational and systemic societal engagements [58]. The technical, and sometimes nontransparent nature of computational modelling research makes it a particularly difficult candidate for meaningful involvement of publics, yet its increasingly significant role in contemporary governance also makes such efforts vital.

## Supplementary Information

Additional file 1.

## Data Availability

Data is available in supplementary information.
