# Supplementary material for: Towards socially robust policy modelling: scoping review of public involvement in computational policy modelling
Source: Health Res Policy Syst. 2026 Mar 11;24:28. doi: 10.1186/s12961-026-01473-6 (PMC13023115; doi:10.1186/s12961-026-01473-6)
Supplement: Supplementary file 1 — Additional file 1. [file 12961_2026_1473_MOESM1_ESM.docx]

| **Review identifier** | **Author(s)** | **Year** | **Title** | **Topic modelled** | **Region** |
| --- | --- | --- | --- | --- | --- |
| 2 | Angelopoulos et al | (2017) | Risk- based analysis and policy implications for renewable energy investments in Greece | Environmental | Europe |
| 23 | Catenacci and Giupponi | (2013) | Integrated assessment of sea-level rise adaptation strategies using a Bayesian decision network approach | Environmental | Europe |
| 1 | de Carvalho et al | (2010) | Application description and policy model in collaborative environment for sharing of information on epidemiological and clinical research data sets | Science policy | North America |
| 7 | Fitzgerald et al | (2018) | Exploring the impact of public health  teams on alcohol premises licensing in  England and Scotland (ExILEnS): procotol  for a mixed methods natural experiment  evaluation | Health | Europe |
| 25 | Freebairn et al | (2019) | Turning conceptual systems maps into  dynamic simulation models: An Australian  case study for diabetes in pregnancy | Health | Australasia |
| 12 | Gupta et al | (2018) | Multi-stakeholder policy modeling for  collection and recycling of spent portable  battery waste | Environmental | Asia |
| 26 | Halbe et al | (2018) | A methodological framework to support the initiation, design and institutionalization of participatory modeling processes in water  resources management | Environmental | North America |
| 18 | Holtgrave et al | (2016) | Using Resource Allocation Modeling to Inform HIV Prevention Priority Setting for Baltimore-Towson, Maryland | Health | North America |
| 27 | Inam et al | (2017) | Coupling of a distributed stakeholder-built system dynamics  socio-economic model with SAHYSMOD for sustainable soil salinity  management – Part 1: Model development | Environmental | Asia |
| 11 | Islam and Kitazawa | (2013) | Modeling of freshwater wetland management strategies for building the public awareness at local level in Bangladesh | Environmental | Asia |
| 10 | Kenter | (2016) | Integrating deliberative monetary valuation, systems modelling and participatory mapping to assess shared values of ecosystem services | Environmental | Europe |
| 6 | Krämer et al | (2013) | Domain-Specific Languages For Agile Urban Policy Modelling | Planning | Europe |
| 28 | Lane et al | (2011) | Doing flood risk science differently: an  experiment in radical scientific method | Environmental | Europe |
| 8 | Lloyd-Williams et al | (2020) | Evaluating stakeholder involvement in building a decision support tool for the NHS Health Checks: co-producing the WorkHORSE study | Health | Europe |
| 29 | Malard-Adam et al | (2024) | Modelling Indigenous small-scale agriculture and food systems in  Guatemala - Hybrid Bayesian inference for data-poor regions | Emvironmental | Central America |
| 9 | Mandal et al | (2022) | ‘Imperfect but useful’: pandemic  response in the Global South can  benefit from greater use of  mathematical modelling | Health | Asia |
| 13 | Melchior et al | (2024) | Policy Development for Societal  Challenges: The Collaborative Agent  Based Modelling Policy Framework | Environmental | Europe |
| 20 | Nespeca et al | (2023) | A Methodology to Develop  Agent-Based Models for Policy Support  Via Qualitative Inquiry | Environmental | Asia |
| 21 | Özesmi & Özesmi | (2004) | Ecological models based on people’s knowledge:  a multi-step fuzzy cognitive mapping approach | Environmental | Asia/Europe (Turkey) |
| 14 | Persada et al | (2018) | Policy Model of Sustainable Infrastructure  Development (Case Study: Bandarlampung City,  Indonesia) | Planning | Asia |
| 22 | Petersen et al | (2011) | Post-Normal Science in Practice at the Netherlands Environmental Assessment Agency | Environmental | Europe |
| 3 | Pye et al | (2018) | Assessing qualitative and quantitative dimensions of uncertainty in energy modelling for policy support in the United Kingdom | Environmental | Europe |
| 24 | Schenk | (2014) | Using stakeholders’ narratives to build an agent-based simulation of a political process. | Planning | Europe |
| 4 | Scherer and Wimmer | (2013) | Bridging narrative scenario texts and formal policy  modeling through conceptual policy modeling | Environmental | Europe |
| 15 | Thomas et al | (2021) | Rapid adaptive modelling for policy support towards achieving Sustainable  Development Goals: Brexit and the livestock sector in Wales | Environmental | Europe |
| 19 | van Vliet et al | (2010) | Linking stakeholders and modellers in scenario studies: the use of Fuzzy Cognitive Maps as a communication and learning tool | Environmental | Europe |

Table 1: papers included in the scoping review

| **Database** | **Search string** | **Number of results** |
| --- | --- | --- |
| Scopus | ( ( TITLE-ABS-KEY ( "patient*" OR "public*" OR "participant*" OR "consumer*" OR "people" OR "citizen*" OR "client*" OR "lay" OR {service user} OR {service users} OR "stakeholder*" OR "community" ) ) )  AND ( ( TITLE-ABS-KEY ( "particip*" OR "involv*" OR "engage*" OR "partner*" OR "collaborat*" OR {advisory group} OR "consult*" OR "PPI" ) ) )  AND ( TITLE-ABS-KEY ( {policy model} OR {policy models} OR {model for government} OR {model in government} OR {model in decision making} OR {model for policy} OR {models in policy} OR {models in decision making} OR {model in decision making} OR {policy making model} OR {policy making models} OR {model in government} OR {models in government} ) ) | 390 |
| Science Citation Index Expanded (SCI_EXPANDED) and Social Science Citation Index (SSCI) via Web of Science | (TS=("policy model*” OR “model* for policy” OR “model* for government” OR “model* in government” OR “model* in decision making”)) AND (LA==("ENGLISH”))  AND (TS=(particip* OR involv* OR engage* OR partner* OR collaborat* OR “advisory group” OR consult* OR PPI)) AND (LA==("ENGLISH”))  AND (TS=(patient* OR public* OR participant* OR consumer* OR people OR citizen* OR client* OR lay OR “service user*” OR stakeholder* OR community)) AND (LA==("ENGLISH”)) | 321 |

Table 2: search strings for database searches
